# Impact of Prehospital Administration of Aspirin and Unfractionated Heparin on In-Hospital Outcomes in Patients with Suspected Myocardial Infarction: A Retrospective Cohort Study

**DOI:** 10.2147/OAEM.S571863

**Published:** 2026-02-20

**Authors:** Wenke Faller, Ioannis Toskas, Diana Heurich, Manuel Sigle, Meinrad Gawaz, Michal Droppa, Karin Anne Lydia Müller, Andreas Goldschmied

**Affiliations:** 1 Department of Cardiology and Angiology, University Hospital Tübingen, Tübingen, Germany

**Keywords:** Prehospital ACS, myocardial infarction, heparin, acetylsalicylic acid

## Abstract

**Purpose:**

In a prehospital setting with limited diagnostic resources, identification of myocardial infarction (MI) can be challenging. However, high diagnostic accuracy of patients with MI is not only necessary to provide early revascularization but also essential to guide prehospital pharmacological therapy. Even though prehospital use of acetylsalicylic acid (ASA) and anticoagulation therapy is widely established, guidelines only recommend these medications in confirmed cases undergoing PCI. This study examines if prehospital treatment with ASA and unfractionated heparin (UFH) influences in-hospital mortality and bleeding rates in patients with suspected MI.

**Patients and Methods:**

In this retrospective, single-center cohort study, prehospital treatment with ASA and UFH in 2756 patients with suspected MI was analyzed. Associations between ASA/UFH and death/bleeding until discharge were investigated. To adjust for possible confounders, multiple logistic regression was performed. Furthermore, stepwise logistic regression was carried out in order to investigate factors that influence emergency physicians (EPs) decision to treat with ASA and UFH.

**Results:**

Prehospitally administered ASA and UFH was not associated with a significant change in mortality (odds ratio [OR], 0.813; 95% confidence interval [CI] 0.453 to 1.461; *p*=0.489 for ASA and OR 1.036; CI 0.566 to 1.898; *p*=0.908 for UFH) or bleeding (OR, 1.142; 95% CI 0.762 to 2.615; *p*=0.273 for ASA and OR 1.053; CI 0.558 to 1.986; *p*=0.874 for UFH). Several factors including the presence of ST elevations, atypical chest pain, and concomitant medication were found to influence the EPs decision to treat with ASA and UFH.

**Conclusion:**

Prehospital administration of ASA and UFH did not affect in-hospital mortality and bleeding outcomes in a cohort of patients with suspected MI. These findings suggest that routine prehospital anticoagulation in suspected MI may not improve short-term outcomes and should be reconsidered pending randomized evidence.

## Introduction

### Background

Chest pain is one of the most common reasons for consultation in emergency medicine.^1^ In a prehospital setting with limited diagnostic resources, identification of myocardial infarction (MI) can be challenging. This diagnostic challenge is critical because chest pain can be the herald of various catastrophic conditions, including aortic dissection and myocardial rupture, which also presents a significant risk of sudden death and requires a vastly different treatment pathway.^2^,^3^ Therefore, high diagnostic accuracy and avoidance of misclassification is not only necessary to provide early, correct therapy but also to avoid causing harm by administering contraindicated medications like unfractionated heparin (UFH) and acetylsalicylic acid (ASA). Due to the feasibility and ethical challenges in the emergency settings and the resulting lack of randomized prospective data, retrospective analyses remain an appropriate tool for generation of scientific evidence.

### Importance

ASA has long been established as a cornerstone in the pharmacologic treatment of patients with MI and improves outcomes. However, the optimal timing of treatment remains unclear with observational studies producing different results and randomized trials did not distinguish between pre and in-hospital treatment.^4–7^

While undoubtedly indicated during percutaneous coronary interventions (PCI), prehospital treatment with UFH has not shown to improve outcomes in patients with non-ST-elevation myocardial infarction (NSTEMI).^8^ However, treatment on first medical contact versus administration in the catheter laboratory leads to higher rates of spontaneous coronary reperfusion.^9^ While guidelines give a class 1A recommendation on treatment with both ASA and UFH, this applies only in confirmed cases who are undergoing PCI. Furthermore, the 2020 ESC guidelines specifically emphasize the need to balance the risks of bleeding and ischemia.^10^ This puts emergency physicians (EPs) in the difficult situation of evaluating risks in a patient collective with an uncertain diagnosis and highlights the need for further studies. Hence, our investigation aims to shed light on real-life physician decision-making as well as ASA and UFH treatment effects using multiple logistic regression models.

### Goals of This Investigation

In this study, we retrospectively analyzed prehospital treatment with ASA and UFH in 2756 patients with suspected MI.

The primary endpoint was to investigate the effect of prehospital treatment with ASA and UFH on in-hospital mortality and bleeding.

The secondary endpoint was the identification of factors associated with prehospital treatment initiation and prediction of emergency physician ASA and UFH treatment.

## Materials and Methods

### Study Design and Setting

In this retrospective, monocentric cohort study, we analyzed prehospital and in-hospital records of patients with a prehospital suspect of MI. Emergency physician (EP) treatment protocols by two locally collaborating rescue stations for emergency medicine admitting their patients to the Emergency Department of the University Hospital Tübingen, Germany, were filtered for diagnoses “STEMI”, “ACS” and “chest pain” between August 2016 and October 2020. All cases that did not meet exclusion criteria (age below 18 years, prehospital death, ambulatory treatment, patient declined transportation, transportation to a medical facility other than the University Hospital, Tübingen, Germany) were included in the study. This yielded a study cohort comprising 2756 patients.

In the German system, whenever an emergency call indicates suspected MI, an emergency physician is automatically dispatched to the scene together with the EMS team. This ensures that a physician is always present on site in such cases. Prehospital emergency physicians have to undergo a Germany-wide standardized training program including theoretical and practical lessons. Local treatment protocols suggest administration of 150–300mg of ASA and 60U/kg of UFH in suspected MI.

### Ethics Approval

The study complies with the declaration of Helsinki and good clinical practice guidelines^11–13^ and was approved by Ethics Committee at the Medical Faculty of the Eberhard Karls University and at the University Hospital of Tübingen (project number 076/2021B02).

### Selection of Participants and Measurements

In all cases, patients were prehospitally treated by an emergency physician and brought to the Emergency Department of the University Hospital Tübingen, Germany. Relevant prehospital patient data like history, ECG interpretation and administered medication was transferred from the prehospital treatment protocols. Medication dose and timing of administration was at the emergency physician’s discretion with UFH dose ranging from 2500 to 10000 units and ASA dose ranging from 100 to 1000mg. All treatment protocols were exclusively carried out by the attending emergency physician and transferred manually to our database by a clinical study investigator. Protocols only allow the prehospital emergency physician to choose between multiple prespecified options whereof “STEMI”, “ACS” and “chest pain” are those associated with MI. Since information on data like suspected diagnoses and prehospital medical treatment is mandatory and clear documentation is enabled by using unique codes additionally to written medication and diagnosis, high data quality was ensured.

In-hospital data was taken retrospectively from our clinical database. This was also exclusively done manually by the clinical study investigators. Since our hospital introduced high-sensitivity troponin essays during the study period, either troponin or high-sensitivity troponin values were taken. To ensure high quality of in-hospital data, information on definitive diagnosis as well as in-hospital mortality and bleeding was taken from the discharge letter which is present in every patient and drafted by the attending consultant cardiologist. Myocardial infarction was defined according to the 2017 and 2020 ESC guidelines.^10^,^14^ STEMI was defined according to ECG changes mentioned in the guidelines (New ST elevation at the J-point in at least two contiguous leads: ≥2.5 mm in men <40 years, ≥2 mm in men ≥40 years, or ≥1.5 mm, in women regardless of age in leads V2–V3 and/or ≥1 mm in the other leads) as well as angiographic confirmation of coronary artery blockage showing TIMI 0 or 1 flow. In-hospital mortality was defined as mortality until discharge, while any bleeding event (BARC II–V) until discharge qualified as in-hospital bleeding.^15^

### Statistical Analysis

Data was analyzed using SPSS and Python. Since data showed no normal distribution (tested using the Kolmogorov Smirnov test), continuous variables are shown as medians with interquartile range whereas dichotomous variables are presented as frequencies with valid percentages. Kruskal–Wallis tests were used to compare metric variables, whereas chi-square tests were used to compare dichotomous variables.

To identify predictors for prehospital treatment with ASA and UFH, multiple logistic regression was used in a forward stepwise selection mode, with *p* < 0.01 as a criterion for staying in the model. The following variables were tested: Time since onset of symptoms, ECG ST depression, ECG inverted T wave, ECG left bundle branch block, ECG sinus rhythm, upper abdominal pain, dyspnea, diaphoresis, initial systolic blood pressure, initial heart rate, initial oxygen saturation, concomitant ASA, concomitant P2Y12-Inhibitors, ECG ST elevation, sex, chest pain (typical), chest pain (atypical), concomitant oral anticoagulation, age and pain intensity. There were no missing data for these variables.

In order to optimize predictive power for UFH and ASA treatment, logistic regression models for those variables with crude p-values <0.01 were established. After scaling of variables, the dataset was randomly split into a training (80%) and internal validation (20%) dataset following standard ML methodology. Within the training loop of the nested cross-fold validation approach, the binary classifiers were trained including hyperparameter tuning and 10-fold cross-validation. Subsequently, the trained classifiers were applied to previously unseen data of the validation dataset (20% of population) in order to avoid overfitting. Following the TRIPOD-AI guidelines, classification metrics were reported and calibration was evaluated. The models were implemented in Python using scikit-learn package (v1.6.1).

Variance inflation factors were calculated in Python using the statsmodels package (v0.14.4).

To adjust for possible confounders while exploring associations between prehospital treatment with ASA/UFH and in-hospital mortality/bleeding, multiple logistic regression was performed. Tested variables included the following: age, cardiac arrest, pain intensity, prehospital nitroglycerine, prehospital akrinor, prehospital catecholamines, prehospital morphine, arterial hypertension, diabetes mellitus, chronic kidney disease, malignancy, positive Troponin I, left ventricular ejection fraction, acute coronary occlusion, coronary intervention and definitive diagnosis. Analyses were carried out for each medication and endpoint separately. A *p*-value < 0.05 was defined as statistically significant for all tests. Power analyses revealed a statistical power of 0.94 for detecting a small possible effect (f^2^=0.01) on mortality.

Missing values for variables used in the logistic regression analysis described above (2850 of 93704 total values; 3.04%) were imputed using multiple stochastic number regression imputations. This way, 5 datasets were generated. For statistical analysis, means of these 5 datasets were used in continuous variables while the modes were used for dichotomous variables.

To calculate classification metrics for prehospital diagnostic accuracy of identifying ST-elevation myocardial infarction (STEMI), suspected prehospital and definitive in-hospital diagnoses were used. Sensitivity was calculated by dividing true positives by true positives plus false negatives, while specificity was calculated by dividing true negatives by true negatives plus false positives. Positive predictive value was calculated by dividing true positives by true positives plus false positives, while negative predictive value was calculated by dividing true negatives by true negatives and false negatives.

### Visualization

SPSS, Microsoft Power Point and online tool “SankeyMATIC” were used to generate figures. Version numbers and producers of all used programs can be found in Supplementary Table 1.

## Results

### Baseline Characteristics Vary Considerably Between Different Suspected Diagnoses

Full baseline characteristics in which patients are stratified to prehospital suspected diagnoses are demonstrated in Table 1. Patients with suspected diagnoses of chest pain were younger and less likely male when compared to the other groups. Except for mineralocorticoid receptor antagonist, patients in the group of ACS had higher likelihoods of concomitant cardiac medication. Unsurprisingly, patients in the STEMI cohort demonstrated higher likelihood of elevated blood concentrations of troponin and CRP. Furthermore, they showed a higher prevalence of elevated aPTT which probably can be attributed to more prehospital treatment with UFH in this cohort. Individuals in this group were also more likely to receive ASA, opiates, P2Y12 inhibitors and vasopressors. They reported the highest pain levels and were accompanied to the hospital by the emergency physician most often. Interestingly, cardiac risk factors were most prevalent in the ACS group except for smoking.

**Table 1:** Baseline Characteristics of Patient Population Stratified by Suspected Prehospital Diagnosis

Parameters	All Patients, N=2756 (100%)	STEMI, N=362 (13.1%)	ACS, N=2024 (73.4%)	Chest Pain N=370 (13.4%)	*p*
Clinical characteristics					
Age	70 (23)	67 (22)	72 (21)	59.5 (35)	**<0.001**
Gender (male)	1615 (58.6)	245 (67.7)	1174 (58.0)	196 (530)	**<0.001**
*Concomitant cardiac medication*					
OAC	552 (20.9)	30 (9.2)	455 (23.2)	66 (18,5)	**<0.001**
ASA	919 (34.8)	82 (25.2)	763 (39.0)	74 (20.8)	**<0.001**
Platelet aggreg.-I.	413 (15.7)	35 (10.7)	354 (18.1)	24 (6.7)	**<0.001**
ß-Blockers	1285 (48.7)	118 (36.0)	1053 (53.8)	114 (32.0)	**<0.001**
ACE-I	863 (32.7)	78 (23.9)	692 (35.4)	93 (26.1)	**<0.001**
ARB	602 (22.8)	69 (21.2)	484 (24.8)	49 (13.8)	**<0.001**
Diuretics	724 (27.4)	60 (18.3)	578 (29.6)	86 (24.2)	**<0.001**
MRA	277 (10.5)	34 (10.4)	212 (10.8)	31 (8.7)	0.483
*Biomarkers*					
NT-proBNP (>300 ng/l)	231 (79.9)	49 (84.5)	158 (79.4)	24 (75.0)	0.530
Hb (<14 g/dl)	1874 (68.6)	212 (59.2)	1423 (70.8)	239 (65.7)	**<0.001**
aPTT (>40 s)	1302 (48.0)	258 (73.1)	1015 (50.7)	29 (8.0)	**<0.001**
TnI (>0,04 µg/l)	551 (38.0)	123 (71.9)	399 (37.0)	29 (14.5)	**<0.001**
Hs-TnI* (>57/37 µg/l)	434 (35.0)	121 (73.8)	282 (30.8)	31 (19.5)	**<0.001**
AllTnI+	985 (36.6)	244 (72.8)	681 (34.1)	60 (16.7)	**<0.001**
CRP (>0.5 mg/dl)	1051 (38.7)	158 (45.5)	762 (38.0)	131 (36.2)	**<0.017**
*CVRF*					
Arterial hypertension	2098 (76.4)	266 (73.7)	1618 (80.1)	214 (58.8)	**<0.001**
DM	680 (24.8)	86 (23,8)	540 (26.7)	54 (14.8)	**<0.001**
Known CHD	1146 (41.8)	112 (31.0)	944 (46.7)	90 (24.8)	**<0.001**
Smoking	562 (33.7)	104 (43.9)	372 (30.7)	86 (39.8)	**<0.001**
BMI kg/m^2^	26.85 (6.87)	26.87 (7.51)	26.82 (6.48)	27,1 (7.57)	0.762
*Preclinical therapy*					
ASA	1355 (49.2)	294 (81.2)	1038 (51.3)	23 (6.2)	**<0.001**
UFH	1372 (49.8)	302 (83.4)	1047 (51.7)	23 (6.2)	**<0.001**
P2Y12-Inh.	13 (0.5)	10 (2.8)	3 (0.1)	0 (0)	**<0.001**
Opiates	1173 (42.6)	217 (59.9)	907 (44.8)	49 (13.2)	**<0.001**
Nitro	922 (33.5)	113 (31.2)	754 (37.3)	55 (14.9)	**<0.001**
Vasopressors	94 (3.4)	42 (11.6)	50 (2.5)	2 (0,5)	**<0.001**
*Preclinical data*					
Pain (0–10)	4 (5)	4 (6)	4 (5)	3 (5)	**0.022**
Accompanied by EP	2639 (95.8)	359 (99.2)	1964 (97.0)	316 (85.4)	**<0.001**
Initial systolic BP	150 (40)	140 (40)	150 (40)	150 (40)	**<0.001**
HR	82 (28)	80 (30)	82 (28)	82 (27)	0.082
spO2	97 (3)	97 (4)	97 (3)	97 (3)	**<0.001**

**Notes**: Significant values are in bold. Continuous variables are shown as medians with IQR since they show no normal distribution. Dichotomous variables are presented as frequency with valid percentages. Kruskal–Wallis tests were used for metric variables whereas chi-square tests were used to compare dichotomous variables. *hsTnI has two values since thresholds are sex-depend (>37 µg/l for females and >57 µg/l for males).

**Abbreviations**: ACE-I, angiotensin converting enzyme inhibitor; AllTnI+, all positive troponin values; Aptt, initial activated partial thromboplastin time; ARB, angiotensin receptor blocker; ASA, acetylsalicylic acid; BMI, body mass index; BP, systolic blood pressure; CHD, coronary heart disease; CRP, C-reactive protein, CVRF, cardiovascular risk factors; DM, diabetes mellitus; EP, emergency physician; Hb, haemoglobin; HR, heart rate; MRA, mineralocorticoid receptor antagonist; nitro, nitroglycerine; NT-proBNP, N-terminal prohormone of brain natriuretic peptide; OAC, oral anticoagulation; Platelet aggreg.-I, platelet aggregation inhibitor; P2Y12-Inh, P2Y12-Inhibitor; spO2, oxygen saturation; TnI, troponin I on presentation; hsTnI, high sensitivity troponin on presentation; UFH. unfractionated heparin.

### Suspected Diagnoses Deviate from Definitive Diagnoses but Influence Prehospital Therapy

**Figure 1:**
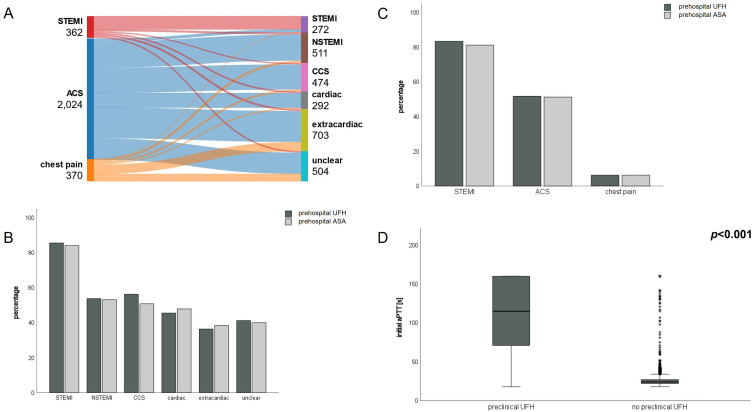
Diagnostic accuracy and preclinical therapy. Sankey plot demonstrating suspected diagnoses (left) and definitive diagnoses (right) of the patient collective (**A**). Bar charts demonstrating UFH and ASA administration by the emergency physician stratified by definitive in-hospital (**B**) and suspected prehospital diagnoses (**C**). Box plots showing activated partial thromboplastin time (aPTT) in patients receiving prehospital UFH (left) or no prehospital UFH (right) (**D**). A Mann–Whitney-*U* test was used to compare median aPTT between groups. The resulting p-value is indicated in the top right corner while asterisks represent extreme values.

**Abbreviations**: STEMI, ST-elevation myocardial infarction; NSTEMI, non-ST-elevation myocardial infarction; ACS, acute coronary syndrome; CCS, chronic coronary syndrome; ASA, acetylsalicylic acid; UFH, unfractionated heparin.

Since prehospital diagnostic modalities are rudimentary and focus on history, examination and ECG only, they deviate considerably from definitive diagnoses at discharge. A sankey plot demonstrating suspected and definitive diagnoses is given in Figure 1A. The biggest heterogeneity can be seen in the large ACS cohort which received a definitive diagnosis ranging from STEMI to extracardiac cause of chest pain. Nevertheless, EPs elected to administer ASA and UFH more often in patients with suspected STEMI (>80%) when compared to ACS (roughly 50%) and chest pain (<10%). This does result in over 80% of patients with a definitive diagnosis of STEMI receiving prehospital therapy with ASA and UFH versus roughly 50% with a definitive diagnosis of NSTEMI and CCS. Calculation of classification metrics for correctly identifying STEMI revealed a sensitivity of 80.9 and a specificity of 94.3% and are demonstrated in Supplementary Table 2. However, roughly 40% of individuals with a definitive diagnosis of extracardiac or unknown cause of symptoms have also been treated. Bar charts visualizing prehospital ASA and UFH treatment stratified to suspected and definitive diagnosis are given in Figure 1B and C.

### A Large Proportion of Patients without Myocardial Infarction Receive Prehospital Anticoagulation Which Could Cause Potential Harm

**Figure 2:**
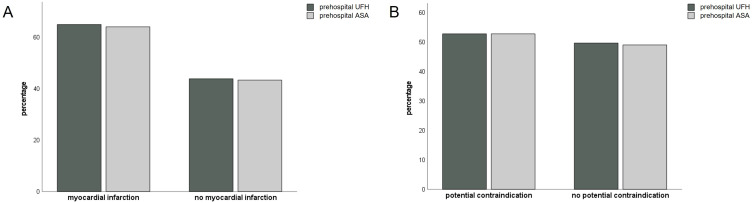
Prehospital therapy stratified by presence of contraindication and myocardial infarction. Bar charts showing percentage of patients prehospitally treated with ASA and UFH. Individuals with an in-hospital diagnosis of myocardial infarction on the left, exclusion of myocardial infarction on the right (**A**). Bar charts demonstrating percentage of patients prehospitally treated with ASA and UFH. Individuals with a potential in-hospital contraindication for anticoagulation are shown on the left, no potential contraindication on the right (**B**).

**Abbreviations**: ASA, acetylsalicylic acid; UFH, unfractionated heparin.

To investigate if prehospital administration of UFH influences measurable blood coagulation metrics, initial aPTT was compared between treated and non-treated patients. Figure 1D shows that mean aPTT is significantly elevated in individuals treated with UFH. Since only patients with myocardial infarction (MI) might benefit from ASA and UFH treatment, we stratified prehospital treatment with these medications to MI and non-MI individuals. Only 64% of patients with MI received ASA and 65% UFH, whereas in individuals with no MI 43% were treated with ASA and 44% with UFH. Bar charts demonstrating these results are given in Figure 2A. Conversely, patients with an in-hospital contraindication for ASA and UFH were investigated. Included contraindications consisted of bleeding, aortic dissection, gastric ulcers, fractures, non-cardiac surgery within 48 hours of admission and platelet levels <30.000/µL. Since these are difficult or impossible to anticipate in a prehospital setting, 53% of patients with a contraindication were treated with ASA and UFH (Figure 2B). However, absolute numbers are relatively small as contraindications were rare in our population (72 cases, 2.6%).

### Prehospital ASA and UFH Do Not Affect Hard Endpoints

Next, multiple logistic regression was used to investigate prehospital therapy with ASA and UFH on bleeding and death. Since this is a retrospective study, the analysis was adjusted for multiple possible confounders listed in the methods section. Of 2756 investigated cases, 87 (3.2%) reached the primary endpoint of in-hospital mortality, whereas 55 had a bleeding event (2%). Neither ASA (*p*=0.489 for mortality, *p*=0.273 for bleeding) nor UFH (*p*=0.908 for mortality, *p*=0.874 for bleeding) had a significant effect on either endpoint (Table 2).

**Table 2:** Prehospitally Administered Anticoagulation in Relation to In-Hospital Mortality and Bleeding Adjusted for Multiple Confounders

	Crude Values			Adjusted Values		
	OR	(95% CI)	*p*	OR	(95% CI)	*p*
In-hospital mortality						
Prehospital UFH	1.033	(0.674,1.584)	0.881	1.036	(0.566, 1.898)	0.908
Prehospital ASA	0.964	(0.629,1.478)	0.866	0.813	(0.453, 1.461)	0.489
In-hospital bleeding						
Prehospital UFH	1.128	(0.661,1.925)	0.659	1.053	(0.558, 1.986)	0.874
Prehospital ASA	1.449	(0.844, 2.489)	0.179	1.142	(0.762, 2.615)	0.273

**Abbreviations**: OR, odds ratio; CI, confidence interval; ASA, acetylsalicylic acid; UFH, unfractionated heparin.

### Decision to Treat with ASA and UFH Depends on Multiple Variables and is Difficult to Predict

**Figure 3:**
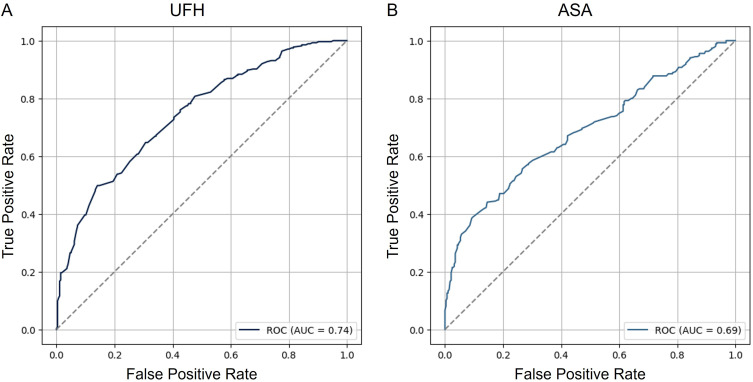
Performance of model prediction for prehospital treatment with UFH and ASA. ROC curves visualizing the false positive rate (1-specificity) on the x-axis and the true positive rate (sensitivity) on the y-axis for prediction of treatment with UFH (**A**) and ASA (**B**).

**Abbreviations**: ASA, acetylsalicylic acid; UFH, unfractionated heparin; ROC, receiver operating characteristic; AUC, area under the curve.

To investigate which variables influence emergency physicians with regard to their decision to administer UFH and ASA in the prehospital setting, logistic regression as described in the methods section was carried out. Unsurprisingly, ST elevations were the strongest predictor for treatment with UFH (OR 3.834; CI 2.991 to 4.915) and ASA (OR 3.619; CI 2.846 to 4.602). Furthermore, increased age and preexisting oral anticoagulation led to more administration of UFH and ASA while presence of atypical chest pain decreased the likelihood of treatment. Significant variables predicting prehospital therapy are listed in Table 3 and Table 4 while a list of all tested variables is demonstrated in the methods section. Area under the receiver operator characteristics (AUROC) curves for prediction of treatment with UFH (A) and ASA (B) is displayed in Figure 3. Even though overall performance was limited, the model for UFH (0.74) outperformed prediction of ASA (0.69) treatment. Additionally, AUROC curves for the training data are demonstrated in Supplementary Figure 1 while calibration curves are displayed in Supplementary Figure 2. Calibration metrics are presented in Supplementary Table 3.

**Table 3:** Predictors for the Use of UFH Prior to Hospital Admission

	Crude Values			Adjusted Values			
	OR	(95% CI)	*p*	OR	(95% CI)	*p*	VIF
ECG ST elevation	4.247	(3.362, 5.366)	**<0.001**	3.834	(2.991, 4.915)	**<0.001**	1.074
Sex (male)	1.391	(1.195,1.620)	**<0.001**	1.426	(1.204, 1.689)	**<0.001**	1.066
Chest pain (typical)	1.825	(1.495,2.229)	**<0.001**	1.526	(1.212, 1.921)	**<0.001**	1.171
Chest pain (atypical)	0.234	(0.171,0.319)	**<0.001**	0.241	(0.173, 0.335)	**<0.001**	1.071
Concomitant OAC	0.301	(0.246,0.369)	**<0.001**	0.260	(0.208, 0.326)	**<0.001**	1.440
Age	1.001	(0.996,1.006)	0.618	1.013	(1.008, 1.019)	**<0.001**	1.425
Pain (0–10)	1.116	(1.086,1.147)	**<0.001**	1.125	(1.090, 1.160)	**<0.001**	1.125

**Note**: Significant p-values are presented in bold.

**Abbreviations**: OR, Odds ratio; CI, Confidence interval; OAC, oral anticoagulation; ECG, electrocardiogram; UFH, unfractionated heparin; VIF, variance inflation factor.

**Table 4:** Predictors for the Use of ASA Prior to Hospital Admission

	Crude Values			Adjusted Values			
	OR	(95% CI)	*p*	OR	(95% CI)	*p*	VIF
Concomitant P2Y12-Inhibitors	0.551	(0.444, 0.683)	**<0.001**	0.521	(0.412, 0.661)	**<0.001**	1.112
Concomitant ASA	0.770	(0.658,0.902)	**0.001**	0.698	(0.578, 0.843)	**<0.001**	1.290
ECG ST depression	1.189	(0.934,1.513)	0.159	1.421	(1.101, 1.835)	**0.007**	1.037
ECG ST elevation	4.137	(3.284,5.213)	**<0.001**	3.619	(2.846, 4.602)	**<0.001**	1.074
Sex (male)	1.263	(1.085,1.470)	**<0.003**	1.345	(1.139, 1.589)	**<0.001**	1.066
Chest pain (atypical)	0.278	(0.206, 0.376)	**<0.001**	0.274	(0.200, 0.375)	**<0.001**	1.071
Concomitant OAC	0.668	(0.554,0.806)	**<0.001**	0.540	(0.432, 0.675)	**<0.001**	1.440
Age	1.004	(1.000,1.009)	0.065	1.014	(1.009, 1.020)	**<0.001**	1.425
Pain (0–10)	1.093	(1.064,1.122)	**<0.001**	1.111	(1.079, 1.144)	**<0.001**	1.125

**Note**: Significant p-values are presented in bold.

**Abbreviations**: OR, Odds ratio; CI, Confidence interval; OAC, oral anticoagulation; ECG, electrocardiogram; UFH, unfractionated heparin; VIF, variance inflation factor.

## Discussion

The prehospital management of patients with suspected acute coronary syndrome (ACS) remains a significant challenge in emergency medicine. This study aimed to investigate the impact of prehospital therapy with ASA and UFH on patient outcomes. Our findings, while not demonstrating significant differences in mortality or bleeding rates, raise important questions about current practices and highlight areas for future research.

### Factors Impacting Prehospital Treatment Decisions

The novelty of our study lies in the fact that all patients were seen and treated by a highly trained emergency physician. Regression analyses revealed that numerous clinical and electrocardiographic factors were considered before deciding whether or not to administer prehospital anticoagulation. Besides electrocardiographic variables and pain characteristics and intensity, emergency physicians focused on demographic factors such as age and sex. However, even though several factors predicting treatment in a prehospital setting could be identified, overall predictive performance was rather weak (AUROC around 0.7). This might be due to rather heterogenic treatment decisions. While only 80% of patients with a prehospital suspect of STEMI were treated, around 50% of patients with the unspecific suspected diagnoses of ACS received ASA and UFH. This might be due to limited guidelines in the prehospital setting.

### Challenges in Prehospital Diagnosis of Myocardial Infarction

As mentioned above, our study emphasizes that accurate diagnosis of myocardial infarction (MI) is difficult due to limited diagnostic resources available prehospitally. Unfortunately, other authors support these findings in several studies on prehospital identification of ACS.^16^,^17^ They found that even experienced emergency physicians struggle to accurately identify MI patients based solely on clinical presentation and ECG findings.^16^ This low diagnostic accuracy underscores the complexity of making treatment decisions in a prehospital setting, particularly regarding the administration of antithrombotic therapies like ASA and UFH.

The difficulty in prehospital MI diagnosis stems from several factors. Firstly, the presenting symptoms of ACS can be highly variable and sometimes atypical, especially in certain patient populations such as the elderly, women, and those with diabetes.^18^ Secondly, the 12-lead ECG, while a valuable tool, has limitations in detecting certain types of MI, such as posterior or right ventricular infarctions.^19^ Lastly, the absence of cardiac biomarkers like troponin in the prehospital setting removes a crucial diagnostic element that is well established in hospital-based ACS evaluation.^20^ Even though authors have described an increase in diagnostic performance with prehospital point of care troponin testing,^21^ these tests are unfortunately not yet widely available.

Compared to MI in general, emergency physicians appear to display a superior diagnostic accuracy with regard to prehospital identification of STEMI. This is likely due to the fact that there are clear ECG criteria which can be applied. Since especially treatment with UFH has the best evidence in this scenario and specificity appears to be high, specific recommendations for prehospital treatment only in STEMI might be warranted.^22^

### Impact of Prehospital ASA and UFH Therapy on Patient Outcomes

Several other studies have investigated the impact of prehospital antithrombotic therapy in ACS patients, with mixed results. A large study by Bloom et al examined prehospital UFH administration in STEMI patients and found no significant effect on clinical outcomes or 30-day survival, although it did lead to fewer coronary artery occlusions at initial angiography.^22^ While ASA has been shown to improve outcomes in patients with MI,^7^ a clear benefit for prehospital administration has not been shown.^4–6^

Regarding bleeding risk specifically, our findings align with those of other studies. The ATLANTIC trial, which investigated prehospital vs in-hospital ticagrelor administration in STEMI patients, found no significant difference in major bleeding rates between the two groups.^23^ Similarly, a retrospective analysis by Goldstein et al on out-of-hospital cardiac arrest patients found that prehospital treatment with ASA and UFH was not associated with increased bleeding rates.^24^

However, it is important to note that most of the studies, including ours, have focused on relatively short-term outcomes. Potential long-term effects of prehospital antithrombotic therapy, particularly in patients who ultimately do not have MI, remain less well understood. Additionally, the heterogeneity in study designs, patient populations, and specific antithrombotic regimens makes direct comparisons challenging.

### ASA and UFH are Administered at Similar Rates Despite Different Levels of Evidence

Our study demonstrates similar rates of therapy with regard to ASA and UFH. Interestingly, emergency physicians also consider similar factors when deciding on whether or not to treat in a prehospital setting. When considering the fact that a mortality benefit has only been shown for ASA in this patient population, one would expect higher treatment rates with ASA when compared to UFH. As mentioned above, clear recommendations in the prehospital setting are lacking at the moment. When considering the evidence, we would advocate for future guidelines to prioritize treatment with ASA rather than UFH, especially in non-confirmed MI cases. However, given the lack of evidence in the prehospital setting, even this recommendation would be based purely on the results of the ISIS-2 trial and display a low level of evidence for the prehospital setting.^7^ This highlights the need for a randomized controlled trial comparing prehospital administration of ASA and UFH to placebo with a longer follow-up.

### Future Perspectives

Given the complexity of prehospital ACS diagnosis and the multitude of variables involved (eg ST-elevation in ECG, clinical symptoms, age, pain characteristics and sex), there is growing interest in leveraging artificial intelligence (AI) to support decision-making. Models for prediction of major adverse cardiac events (MACE), in-hospital mortality and 12-lead ECG interpretation have been successfully implemented using only pre-hospital data.^16^,^25^,^26^

The potential advantages of AI-assisted decision support in the prehospital setting are numerous. These systems could integrate and analyze a wide range of clinical data points more quickly and consistently than human operators, potentially improving diagnostic accuracy and treatment decisions. Moreover, AI models could be continuously updated with new data, allowing them to adapt to changing patient populations and clinical practices.^27^

This potential increase in diagnostic precision could benefit patients substantially since our present study revealed poor diagnostic accuracy with regard to prehospital administration of ASA and UFH.

## Limitations

Our study has several limitations. Despite adjusted multivariate analyses, residual confounding by indication remains possible due to treatment allocation (eg, ASA/UFH use) being influenced by baseline disease severity – a limitation observational data cannot fully resolve. Additionally, unmeasured confounding factors inherent to non-randomized studies may persist.

Furthermore, the retrospective design may underestimate bleeding complications since minor events might be underreported. Also, medication adherence and exact dosing could not be verified which may affect outcome measurement.

Since our study was conducted in a single-center setting with physician-staffed prehospital care and a unique dispatch and triage structure, the generalizability of our findings may be limited to similar systems in which emergency physicians routinely operate in the prehospital environment.

Lastly, the endpoints death and bleeding were only tracked until patient discharge which created a very short follow-up.

## Conclusion

Prehospital administered ASA and UFH by an emergency physician was not associated with a significant change in mortality or bleeding in patients with suspected MI. Several factors including presence of ST elevations, atypical chest pain and concomitant medication influence emergency physician’s decision to treat with ASA and UFH. In the prehospital setting, great diagnostic uncertainty forces emergency physicians to carefully balance risks and benefits when treating patients with suspected MI. Given the absence of mortality or bleeding benefit, prehospital ASA and UFH administration should be carefully individualized, particularly in patients without clear STEMI features. Ultimately, randomized prospective trials are required in order to answer these questions definitively and guide prehospital anticoagulation protocols.

## Data Availability

Study data is available from the corresponding author upon reasonable request.

## Abbreviations

MI, myocardial infarction; ACE-I, angiotensin converting enzyme inhibitor; aPTT, activated partial thromboplastin time; ARB, angiotensin receptor blocker; ASA, acetylsalicylic acid; BMI, body mass index; BP, systolic blood pressure; CHD, coronary heart disease; CRP, C-reactive protein; CVRF, cardiovascular risk factors; DM, diabetes mellitus; EP, emergency physician; Hb, haemoglobin; HR, heart rate; MRA, mineralocorticoid receptor antagonist; nitro, nitroglycerine; NT-proBNP, N-terminal prohormone of brain natriuretic peptide; OAC, oral anticoagulation; spO2, oxygen saturation; TnI, troponin I; hsTnI, high-sensitivity troponin; ACS, acute coronary syndrome; PCI, percutaneous coronary interventions; NSTEMI, non-ST-elevation myocardial infarction; UFH, unfractionated heparin; STEMI, ST, elevation myocardial infarction; VIF, variance inflation factor.

## References

[1] Goodacre S, Cross E, Arnold J, Angelini K, Capewell S, Nicholl J. The health care burden of acute chest pain. *Heart*. 2005;91(2):229–12. doi:10.1136/hrt.2003.027599 15657244 PMC1768669

[2] Kanani J, Modi K. A rare case of sudden death due to rupture of saccular descending thoracic aortic aneurysm with dissection. *Heart Views*. 2024;25(4):270–274. doi:10.4103/heartviews.heartviews_100_24 40488148 PMC12139647

[3] Kanani J, Sheikh MI. Ruptured dissecting aorta: an uncommon cause of sudden death—An autopsy study. *Cirugía Cardiovasc*. 2025. doi:10.1016/j.circv.2024.11.009

[4] Bang A, Herlitz J, Grip L, et al. The relative influence of age, previous history and therapeutic strategies prior to hospital admission among ambulance transported patients with ST-elevation myocardial infarction. *Int J Cardiol*. 2009;136(2):213–214. doi:10.1016/j.ijcard.2008.04.014 18639944

[5] Barbash I, Freimark D, Gottlieb S, et al. Outcome of myocardial infarction in patients treated with aspirin is enhanced by pre-hospital administration. *Cardiology*. 2002;98(3):141–147. doi:10.1159/000066324 12417813

[6] Zijlstra F, Ernst N, de Boer M-J, et al. Influence of prehospital administration of aspirin and heparin on initial patency of the infarct-related artery in patients with acute ST elevation myocardial infarction. *J Am Coll Cardiol*. 2002;39(11):1733–1737. doi:10.1016/s0735-1097(02)01856-9 12039484

[7] ISIS-2 Collaborative Group. Randomised trial of intravenous streptokinase, oral aspirin, both, or neither among 17,187 cases of suspected acute myocardial infarction: ISIS-2. ISIS-2 (second international study of infarct survival) collaborative group. *Lancet*. 1988;2(8607):349–360. 2899772

[8] Sundermeyer J, Schock A, Kellner C, et al. Pre-hospital admission of heparin in patients with suspected non-ST segment elevation acute coronary syndrome. *Clin Res Cardiol*. 2024. doi:10.1007/s00392-024-02507-1 PMC12089214 39102002

[9] Chen J, Xu C, Qiu L, et al. Heparin administration at first medical contact vs immediately before primary percutaneous coronary intervention: the HELP-PCI trial. *Eur Heart J*. 2025;46(39):3888–3901. doi:10.1093/eurheartj/ehaf481 40748607 PMC12517749

[10] Collet JP, Thiele H, Barbato E, et al. ESC guidelines for the management of acute coronary syndromes in patients presenting without persistent ST-segment elevation. *Eur Heart J*. 2021;42(14):1289–1367. doi:10.1093/eurheartj/ehaa575 32860058

[11] World Medical Association Declaration of Helsinki. Recommendations guiding physicians in biomedical research involving human subjects. *Cardiovasc Res*. 1997;35(1):2–3. 9302340

[12] Directive 2001/20/EC of the European Parliament and of the Council of 4 April 2001 on the approximation of the laws, regulations and administrative provisions of the member states relating to the implementation of good clinical practice in the conduct of clinical trials on medicinal products for human use. *Med Etika Bioet Spring-Summer*. 2002;9(1–2):12–19. Available from: https://pubmed.ncbi.nlm.nih.gov/16276663/. Accessed February 17, 2026. 16276663

[13] ICH harmonised tripartite guideline: guideline for good clinical practice. 8. Essential documents for the conduct of a clinical trial. *J Postgrad Med*. 2001;47(4):264–267. Available from: https://pubmed.ncbi.nlm.nih.gov/11832645/. Accessed February 17, 2026. 11832645

[14] Ibanez B, James S, Agewall S, et al. 2017 ESC guidelines for the management of acute myocardial infarction in patients presenting with ST-segment elevation. *Rev Esp Cardiol*. 2017;70(12):1082. doi:10.1016/j.rec.2017.11.010 29198432

[15] Vranckx P, White HD, Huang Z, et al. Validation of BARC bleeding criteria in patients with acute coronary syndromes: the TRACER trial. *J Am Coll Cardiol*. 2016;67(18):2135–2144. doi:10.1016/j.jacc.2016.02.056 27151345

[16] Goldschmied A, Sigle M, Faller W, Heurich D, Gawaz M, Müller KAL. Preclinical identification of acute coronary syndrome without high sensitivity troponin assays using machine learning algorithms. *Scientific Reports*. 2024;14(1):ARTN9796. doi:10.1038/s41598-024-60249-6 PMC11058266 38684774

[17] Backus BE, Six AJ, Kelder JC, et al. A prospective validation of the HEART score for chest pain patients at the emergency department. *Int J Cardiol*. 2013;168(3):2153–2158. doi:10.1016/j.ijcard.2013.01.255 23465250

[18] Canto JG, Goldberg RJ, Hand MM, et al. Symptom presentation of women with acute coronary syndromes: myth vs reality. *Arch Intern Med*. 2007;167(22):2405–2413. doi:10.1001/archinte.167.22.2405 18071161

[19] Thygesen K, Alpert JS, Jaffe AS, et al. Fourth universal definition of myocardial infarction (2018). *Circulation*. 2018;138(20):e618–e651. doi:10.1161/CIR.0000000000000617 30571511

[20] Roffi M, Patrono C, Collet J-P, et al.[2015 ESC guidelines for the management of acute coronary syndromes in patients presenting without persistent ST-segment elevation. Task force for the management of acute coronary syndromes in patients presenting without persistent st-segment elevation of the european society of cardiology (ESC)]. *Giornale italiano di cardiologia*. 2016;17(10):831–872. doi:10.1714/2464.25804 27869901

[21] Sagel D, Vlaar PJ, van Roosmalen R, et al. Prehospital risk stratification in patients with chest pain. *Emerg Med J*. 2021;38(11):814–819. doi:10.1136/emermed-2020-210212 34373266 PMC8551969

[22] Bloom JE, Andrew E, Nehme Z, et al. Pre-hospital heparin use for ST-elevation myocardial infarction is safe and improves angiographic outcomes. *Eur Heart J Acute Cardiovasc Care*. 2021;10(10):1140–1147. doi:10.1093/ehjacc/zuab032 34189566

[23] Montalescot G, van ‘t Hof AW, Lapostolle F. Prehospital ticagrelor in ST-segment elevation myocardial infarction. *N Engl J Med*. 2014;371(24):2339. doi:10.1056/NEJMc1412729 25494279

[24] Macherey-Meyer S, Heyne S, Meertens MM, et al. Outcome of out-of-hospital cardiac arrest patients stratified by pre-clinical loading with aspirin and heparin: a retrospective cohort analysis. *J Clin Med*. 2023;12(11):3817. doi:10.3390/jcm12113817 37298012 PMC10253358

[25] Goto S, Kimura M, Katsumata Y, et al. Artificial intelligence to predict needs for urgent revascularization from 12-leads electrocardiography in emergency patients. *PLoS One*. 2019;14(1):e0210103. doi:10.1371/journal.pone.0210103 30625197 PMC6326503

[26] Al-Zaiti S, Besomi L, Bouzid Z, et al. Machine learning-based prediction of acute coronary syndrome using only the pre-hospital 12-lead electrocardiogram. *Nat Commun*. 2020;11(1):3966. doi:10.1038/s41467-020-17804-2 32769990 PMC7414145

[27] Topol EJ. High-performance medicine: the convergence of human and artificial intelligence. *Nat Med*. 2019;25(1):44–56. doi:10.1038/s41591-018-0300-7 30617339

